# Restoration of Limb Length Discrepancy and Alignment With the Ilizarov Device After Management of an Aneurysmal Bone Cyst Crossing the Distal Femoral Physis

**DOI:** 10.7759/cureus.46259

**Published:** 2023-09-30

**Authors:** Nickolaos Laliotis, Panagiotis Konstantinidis, Chrysanthos Chrysanthou, Lamprini Giannakopoulou, Elisavet Papadopoulou

**Affiliations:** 1 Orthopaedics, Interbalkan Medical Center, Thessaloniki, GRC; 2 Anatomy and Surgical Anatomy, Aristotle University of Thessaloniki, Thessaloniki, GRC; 3 Radiology, Interbalkan Medical Center, Thessaloniki, GRC

**Keywords:** distraction osteogenesis, ilizarov, benign bone tumour, growth plate lesion, juxta-physeal, aneurysmal bone cyst

## Abstract

An aneurysmal bone cyst (ABC), when located juxta-physeal, may rarely penetrate the growth plate and extend into the epiphysis. The recurrence rate is considered higher when ABC is in contact with the active growth plate. Treatment methods usually focus on cyst healing and the rate of cyst recurrence. We present the method of treatment used for addressing the lesion of the growth plate following the surgical management and healing of a juxta-physeal ABC. A seven-year-old girl had an aggressive ABC in the juxta-physeal area of the distal femur, penetrating the growth plate and extending in the epiphysis. Surgical treatment was performed, including curettage and autologous bone grafting, avoiding the growth plate. The cyst healed; however, physis presented an obliteration. The affected limb developed valgus deformity and severe leg length discrepancy (LLD). To address this issue, once our patient completed her growth, we proceeded with distraction osteogenesis, using the Ilizarov device, with asymmetrical lengthening of the rods. We achieved the correction of the limb alignment and resolved the LLD. ABCs in the juxta-physeal area of a growing child are benign metaphyseal tumors that exceptionally may penetrate the physis and extend into the epiphysis. Our report highlights that the growth plate's lesion, despite the cyst's healing, may compromise the final result. The use of the Ilizarov device is an effective method for correcting the malalignment and the LLD that may emerge.

## Introduction

Aneurysmal bone cysts (ABCs) are benign neoplasms characterized as expansive osteolytic lesions, predominantly affecting the metaphyseal region of growing children. These tumors are most common in adolescents, constituting approximately 9% of all benign bone tumors. More than 90% of ABCs occur before the age of 20 years. An ABC is usually eccentric, affecting the metaphyseal and diaphyseal areas. Juxtaepiphyseal lesions are reported to be more aggressive and have a higher incidence of recurrence. The growth plate acts as a barrier to the expansion of the cyst, and it is sporadic for a cyst to invade the physis and extend in the epiphysis [1-4]. We have previously described a unique case of a patient with ABC of the distal femoral metaphysis extending in the epiphysis, penetrating the growth plate [5]. During the surgical management of the juxtaepiphyseal cysts with curettage, there is concern for the possible damage to the growth plate. These cysts are more aggressive and may compromise normal physis development. The damage of the open growth plate, mainly in children younger than 12-14 years, may severely affect limb alignment and create leg length discrepancy (LLD). Various treatment methods have been reported for managing ABCs related to the incidence of cyst recurrence [6-8]. There are no previous reports on managing the late sequelae of the growth plate lesion. Herein, we report the long-term outcomes following the initial management of an ABC in a seven-year-old girl, wherein the ABC extended from the distal femoral metaphysis into the epiphysis, crossing the growth plate. After the initial curettage, the cyst did not recur. However, the girl gradually began presenting disturbances in limb alignment, including shortening and limping. By the age of 14, the patient had developed a 21-degree valgus deformity and an LLD of 5 cm. She underwent treatment with distraction osteogenesis using the Ilizarov apparatus, employing asymmetrical lengthening to correct both alignment and length discrepancy. This approach successfully corrected the alignment and mitigated the limb length discrepancy.

## Case presentation

**Figure 1:**
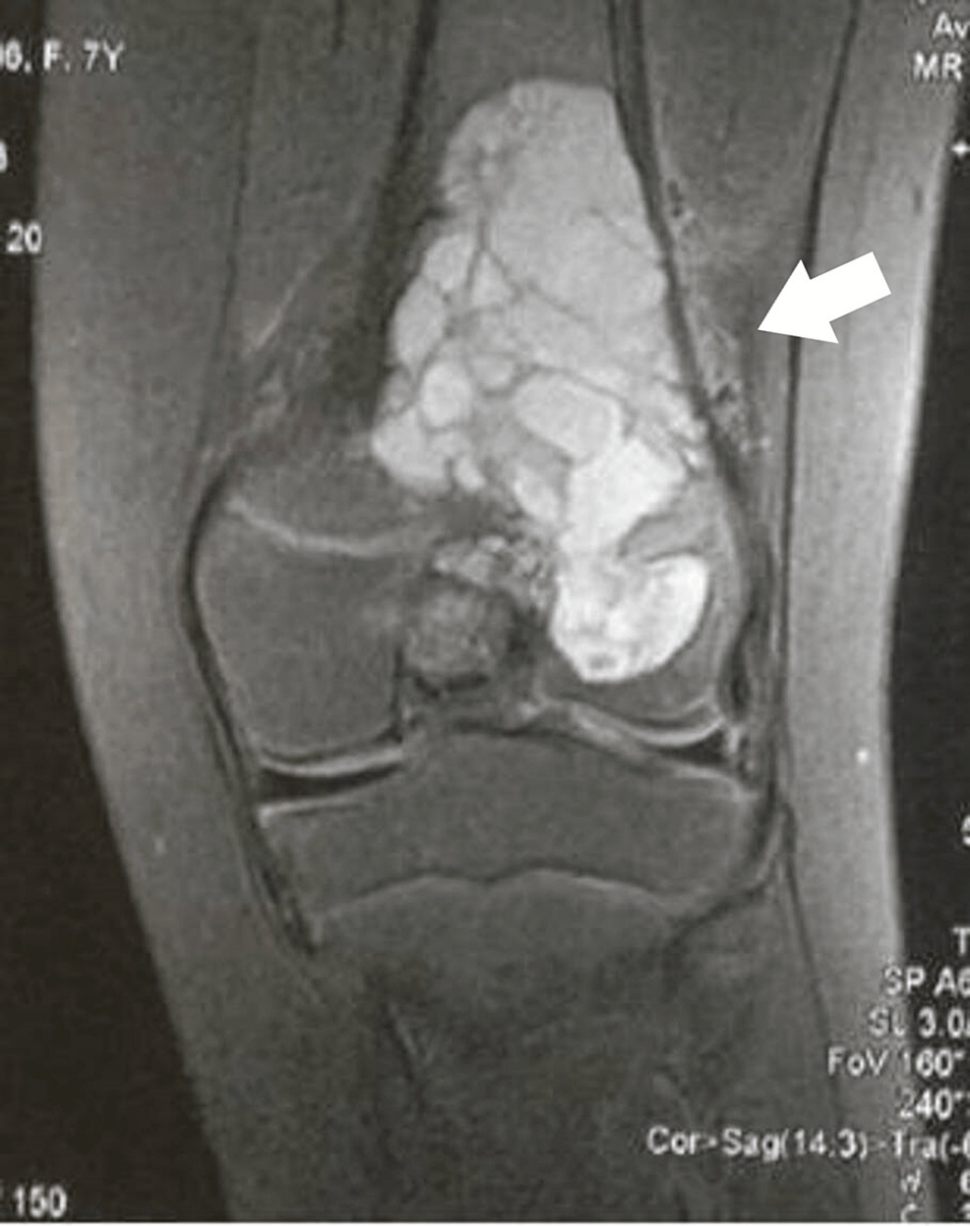
A coronal T1-weighted MRI image showing multiple fluid-fluid levels in the metaphysis, crossing the physis, and extending into the epiphysis.

**Figure 2:**
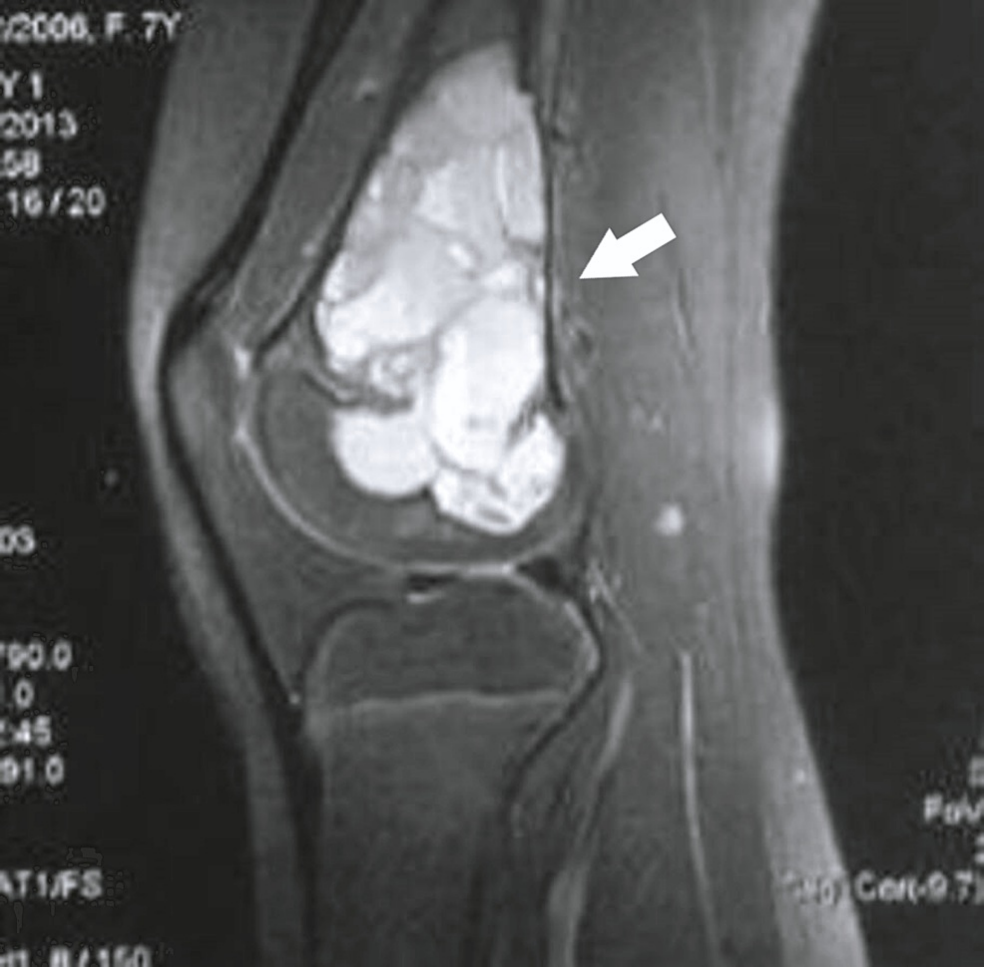
A sagittal T1 FS (Gadolinium-enhanced) weighted MRI image displaying multiple fluid-fluid levels in the metaphysis, traversing the physis and progressing into the epiphysis. Observe the thinning of the posterior cortex and the lack of a periosteal reaction. FS: Fat saturation.

A seven-year-old girl was initially brought to our pediatric orthopedic department for evaluation due to swelling and pain in her left knee. Radiological examinations revealed an eccentric, expansive osteolytic lesion in the distal femoral metaphysis and epiphysis. MRI scans displayed a polylobulated, fluid-containing lesion exhibiting fluid-fluid levels within the metaphysis, crossing the growth plate, and extending into the epiphysis, though not penetrating the articular cartilage. No adjacent soft tissues were involved. The cortices were thinned, with no periosteal elevation observed (Figures 1-2).

**Figure 3:**
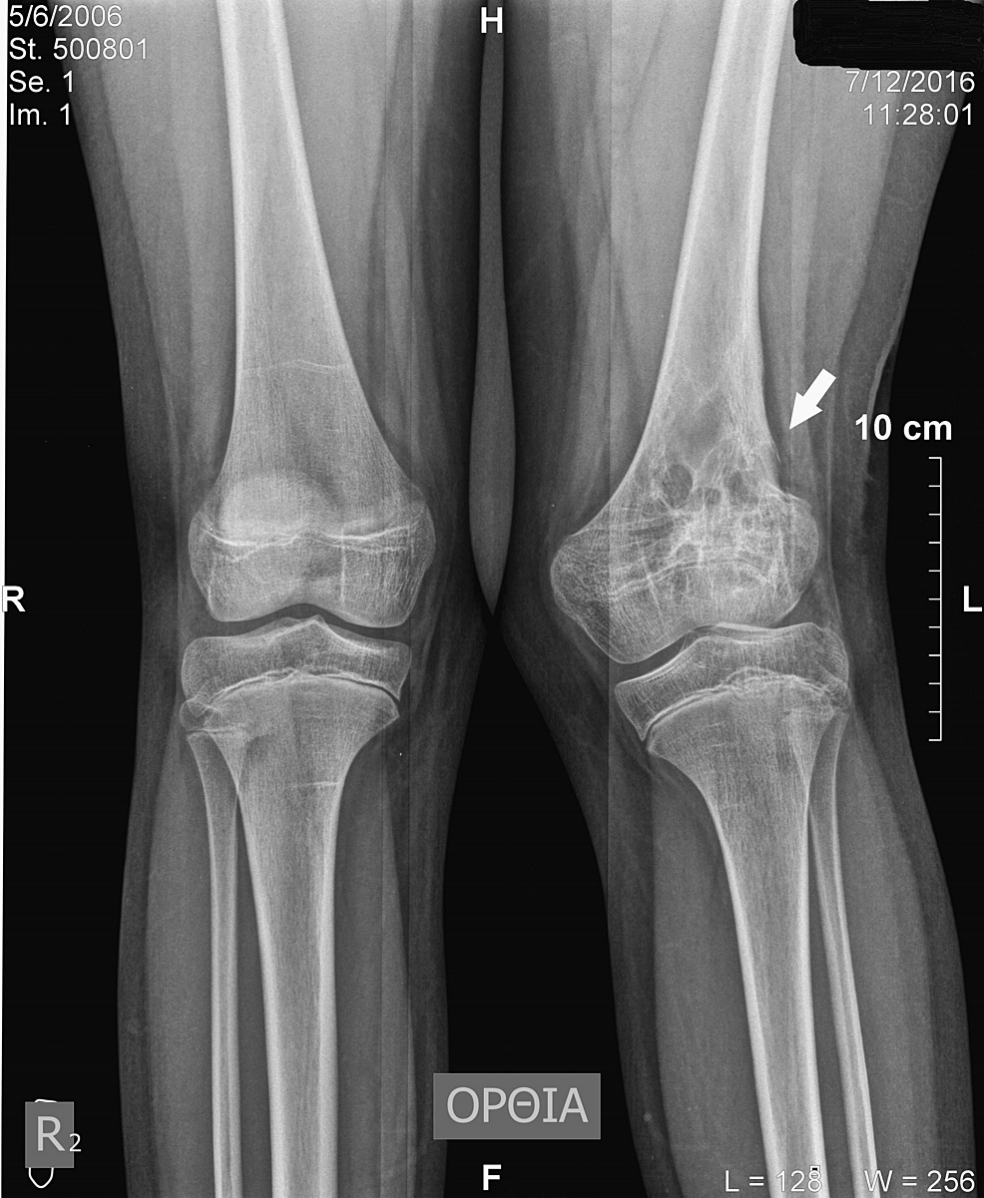
Standing X-ray image illustrates the healing process of the cyst and depicts the restored cortex. A reduced height of the lateral femoral condyle and obliteration of the normal radiolucency of the physis are also observable.

**Figure 4:**
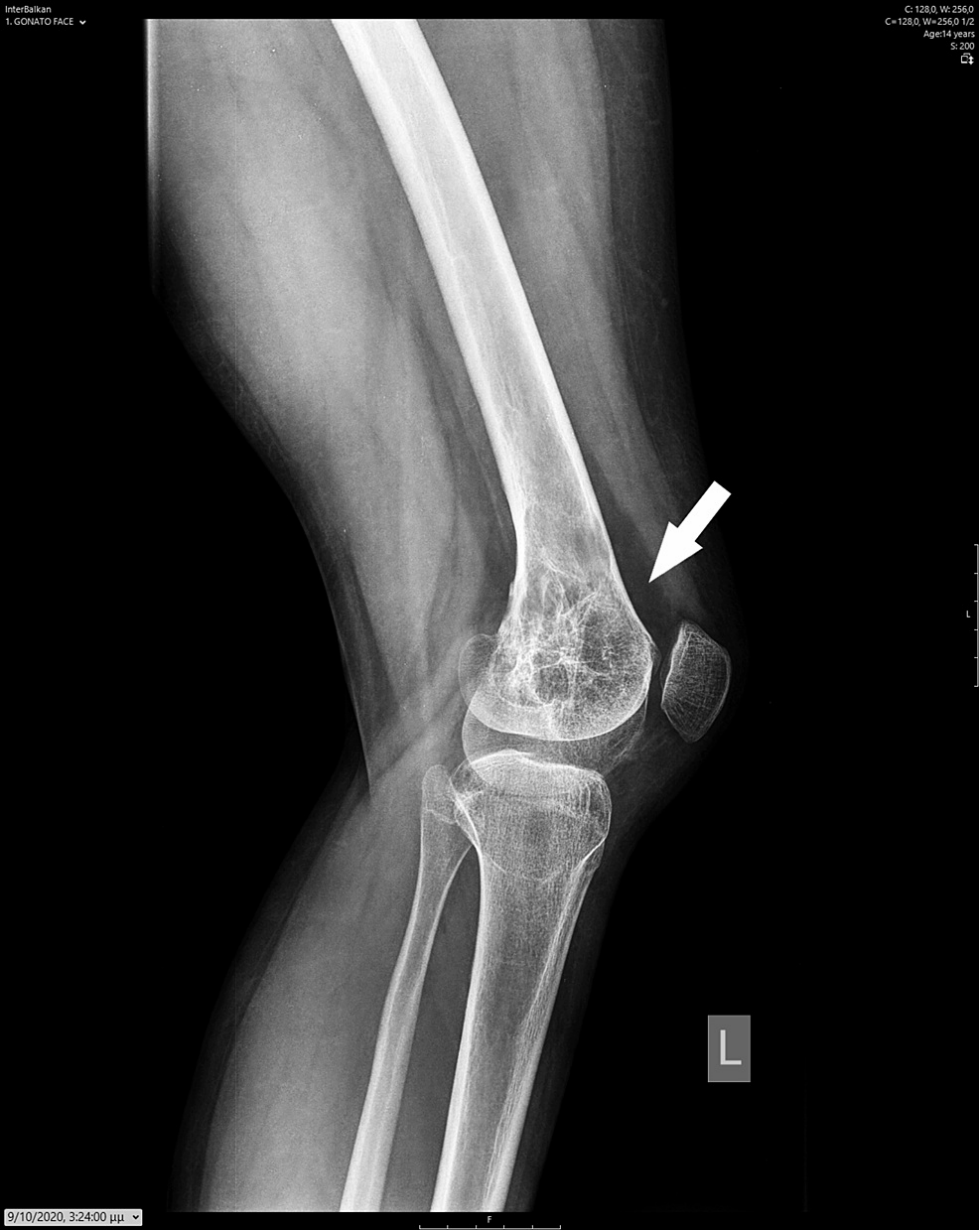
Lateral X-ray image depicts the healed cyst and the restored cortex.

The patient underwent surgery for a thorough lesion curettage, utilizing a lateral approach. A lateral window was opened in the metaphyseal and epiphyseal cortices, carefully avoiding the growth plate area to perform the curettage and to introduce autologous cortical and cancellous bone from the ipsilateral ilium. A notable observation was the complete thinning of the lateral cortices. Pathological examination confirmed the diagnosis of an ABC. The patient experienced an initially uncomplicated recovery, with regular follow-ups every six months. Two years post-operation, she began to exhibit a mild valgus deformity of 15 degrees at the knee joint. Radiological assessments indicated a reduction in the initial radiolucent elements of the cyst, increased sclerosis in the main part of the cyst, and restoration and thickening of the lateral cortices. Additionally, the growth plate exhibited obliteration of the normal radiolucent area from the central to the lateral part (Figures 3-4).

**Figure 5:**
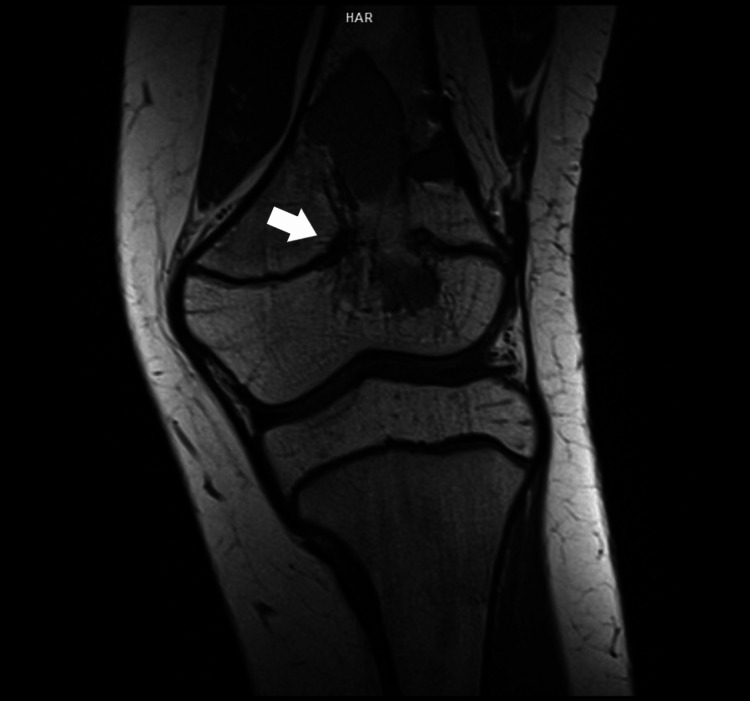
Coronal T1 MRI image displays the diminished diameter of the remaining cyst elements, alongside disturbance of the normal growth plate line.

**Figure 6:**
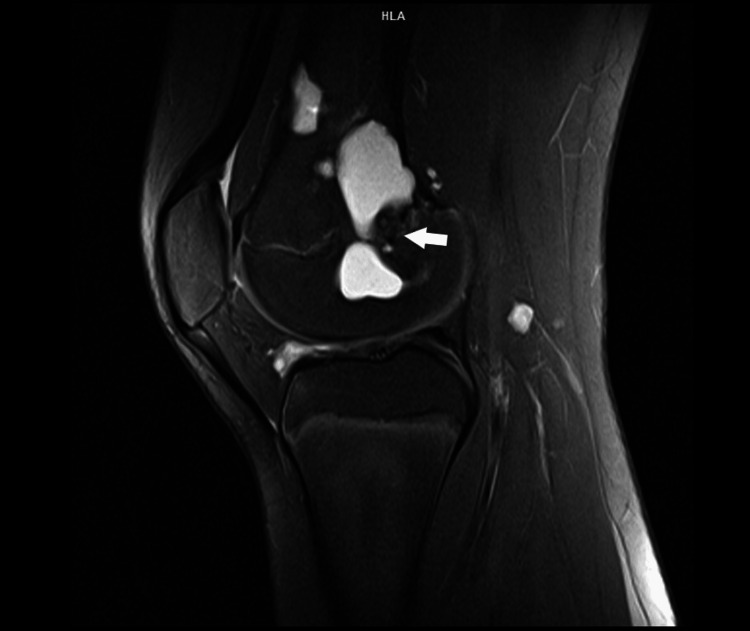
Sagittal T1 FS MRI image displays the reduced diameter of the remaining elements of the cyst, along with the disturbance of the normal line of the growth plate. FS: Fat saturation.

**Figure 7:**
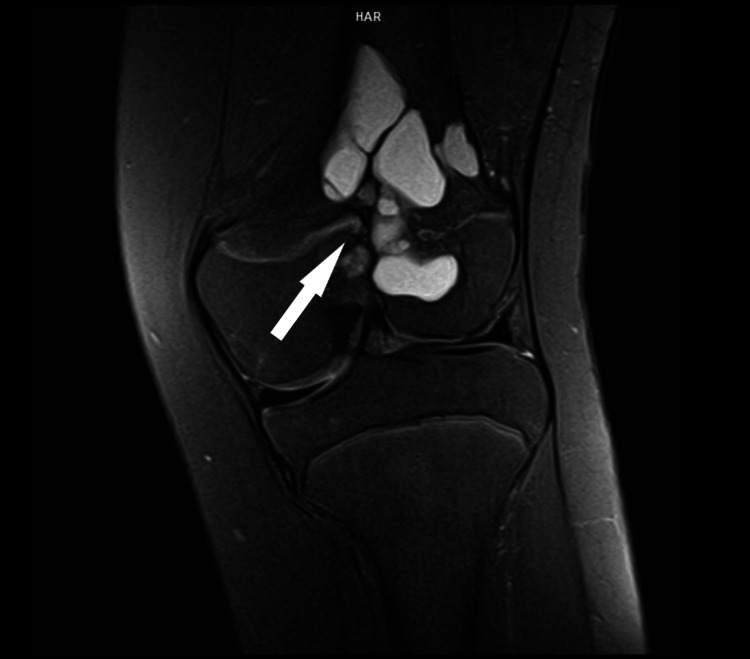
A coronal T1 FS MRI image shows the reduced diameter of the remaining elements of the cyst and illustrates the disturbance to the normal line of the growth plate. FS: Fat saturation.

MRI follow-up showed the absence of tumor recurrence, characterized by important cyst resolution and cortice restoration. The ABC remained with the same volume in the metaphysis and epiphysis, as seen in the postoperative evaluation (Figures 5-7).

**Figure 8:**
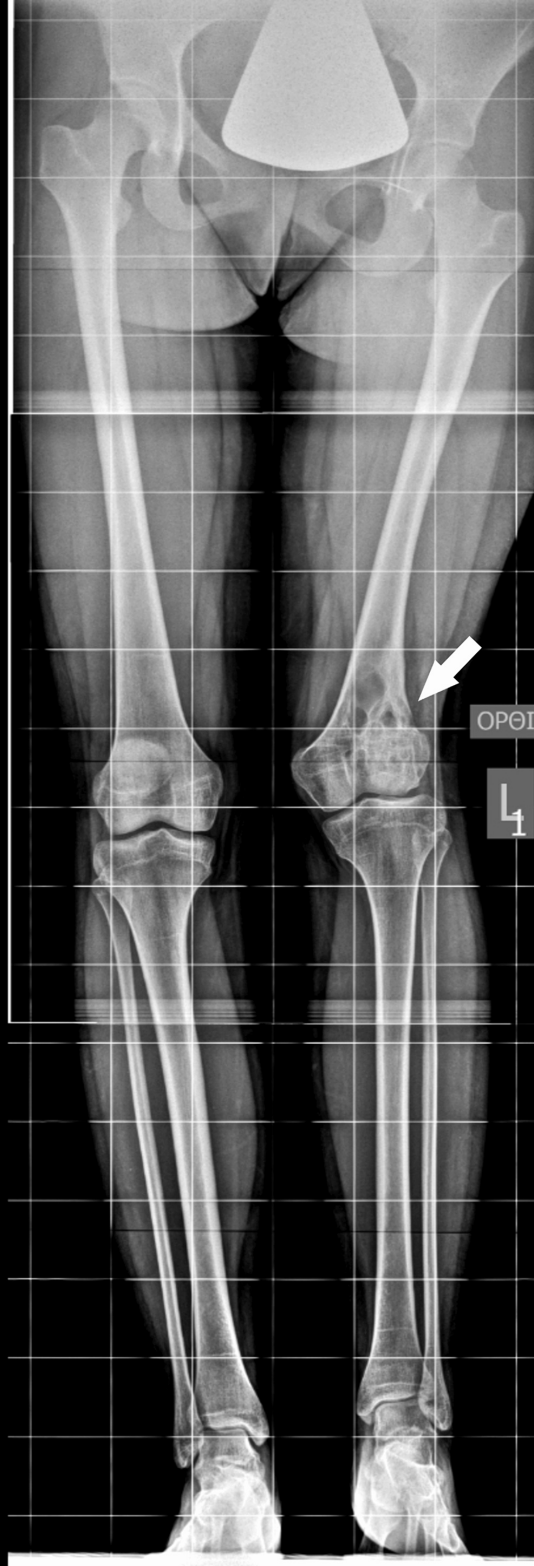
A standing X-ray taken at the end of growth, depicting valgus deformity of the knee and LLD. LLD: Leg length discrepancy.

**Figure 9:**
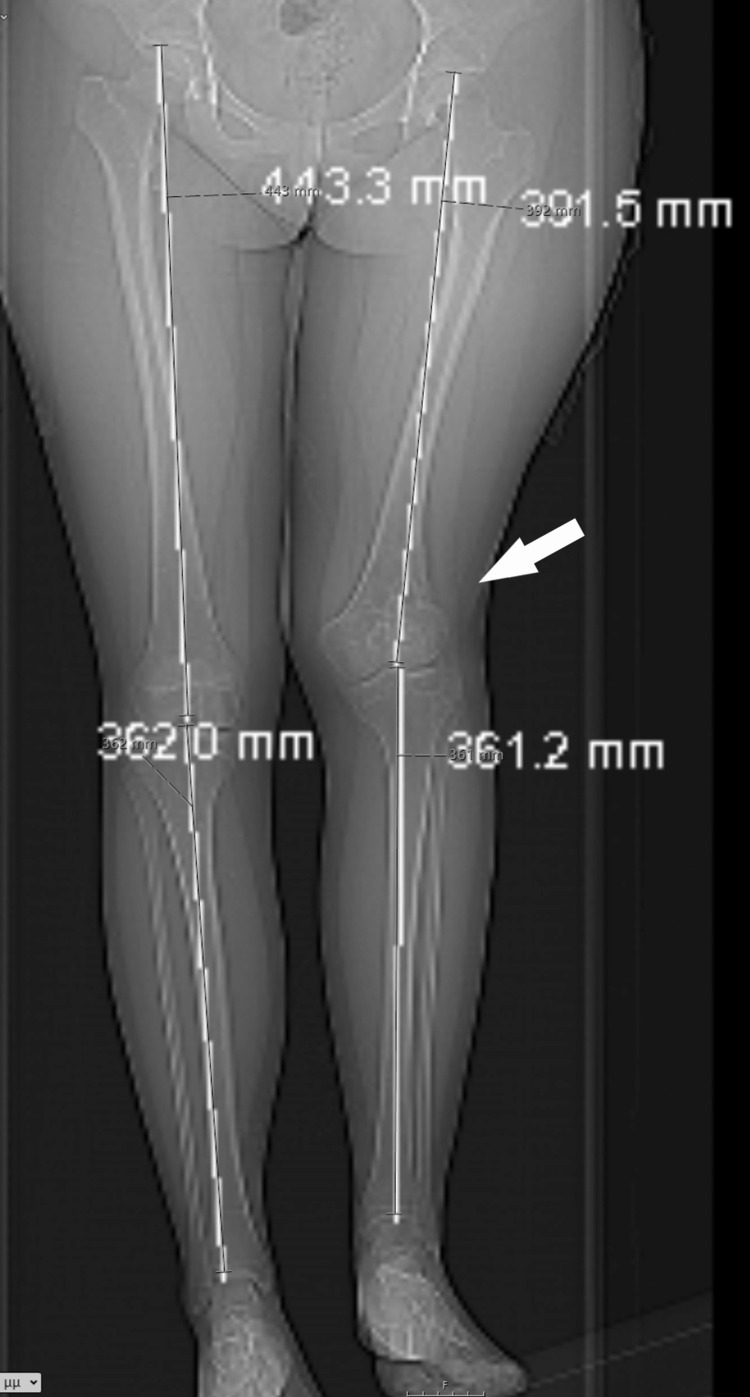
On the patient's scanogram, a 5 cm LLD affecting the femur was measured. LLD: Leg length discrepancy.

At the age of 14 years, the girl displayed a valgus deformity of her knee and an LLD of 5 cm, as measured on a scanogram. There was a reduction in the height of the lateral femoral condyle, resulting in a valgus knee joint line (Figures 8-9).

**Figure 10:**
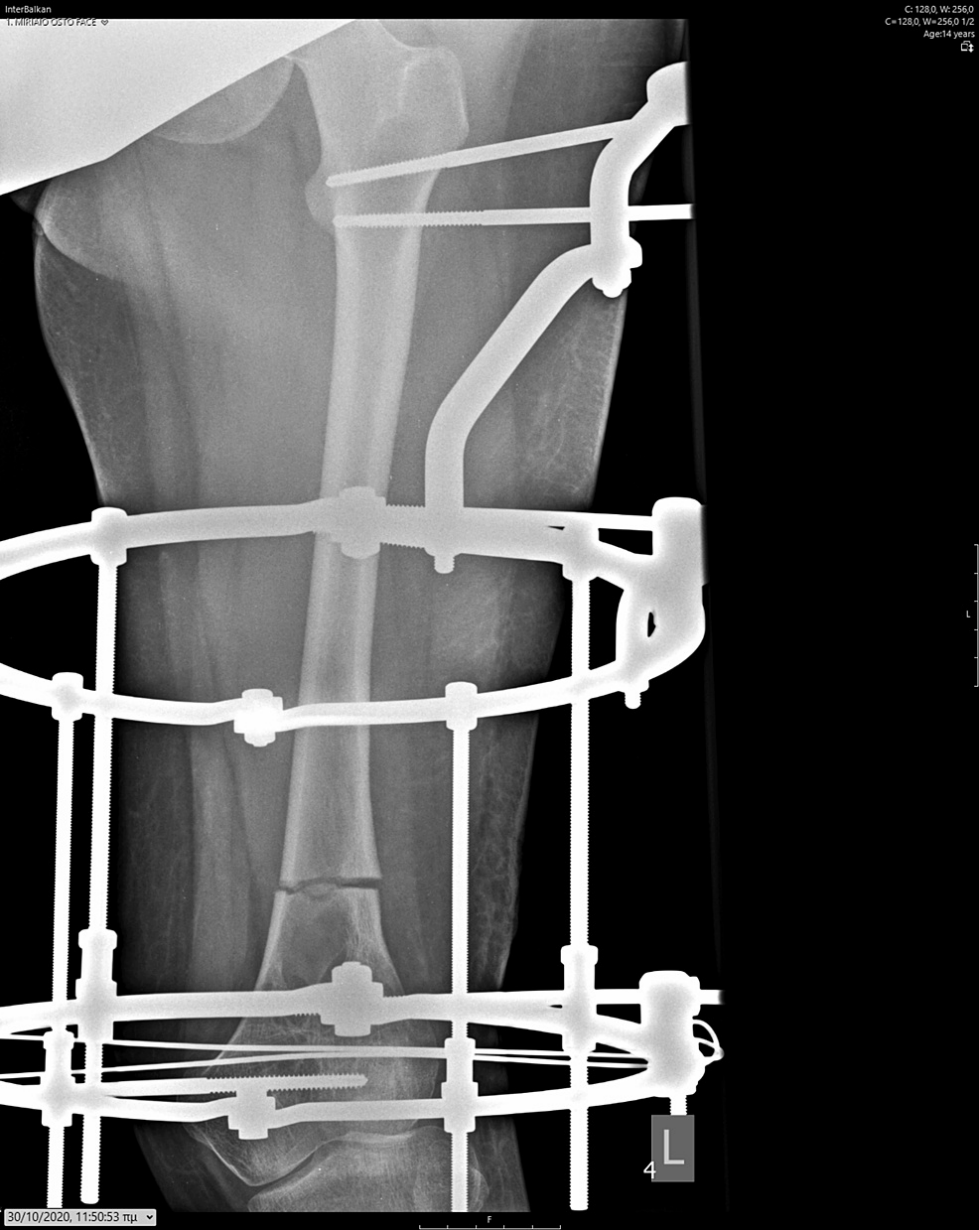
Antero-posterior X-ray illustrating the initial application of the Ilizarov device.

**Figure 11:**
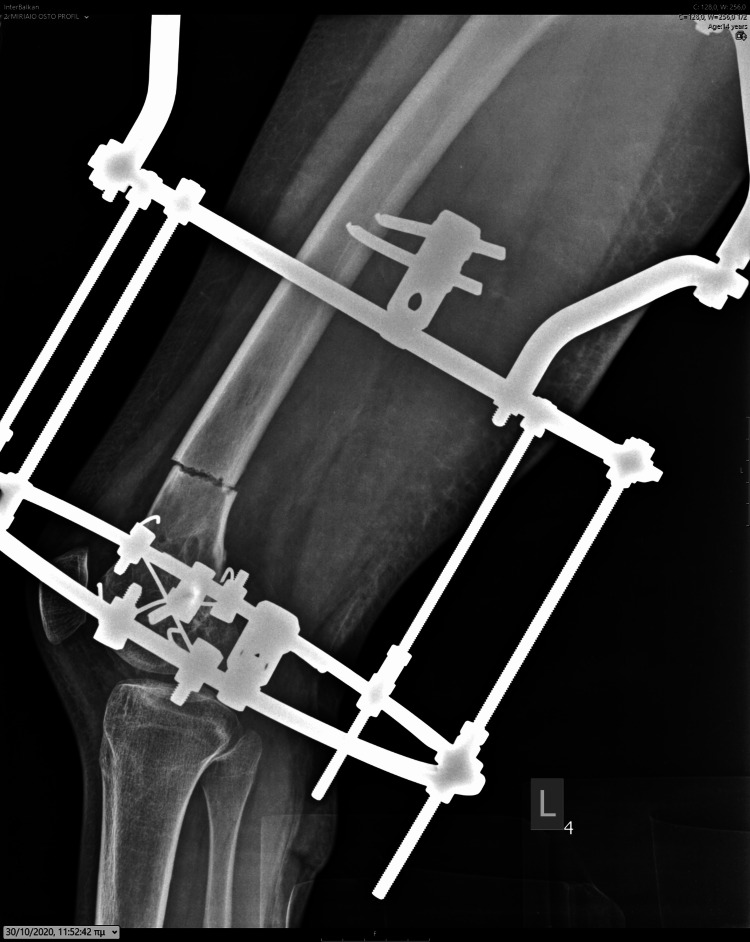
Lateral X-ray illustrating the initial application of the Ilizarov device.

**Figure 12:**
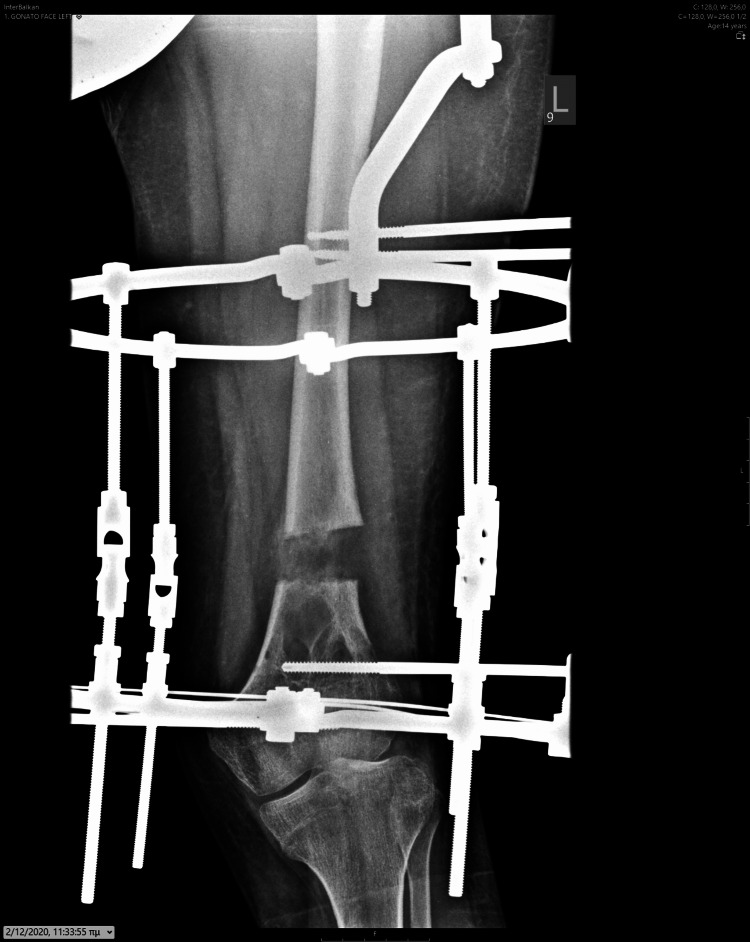
Antero-posterior X-ray of the femur, depicting the asymmetrical lengthening achieved through the Ilizarov device.

**Figure 13:**
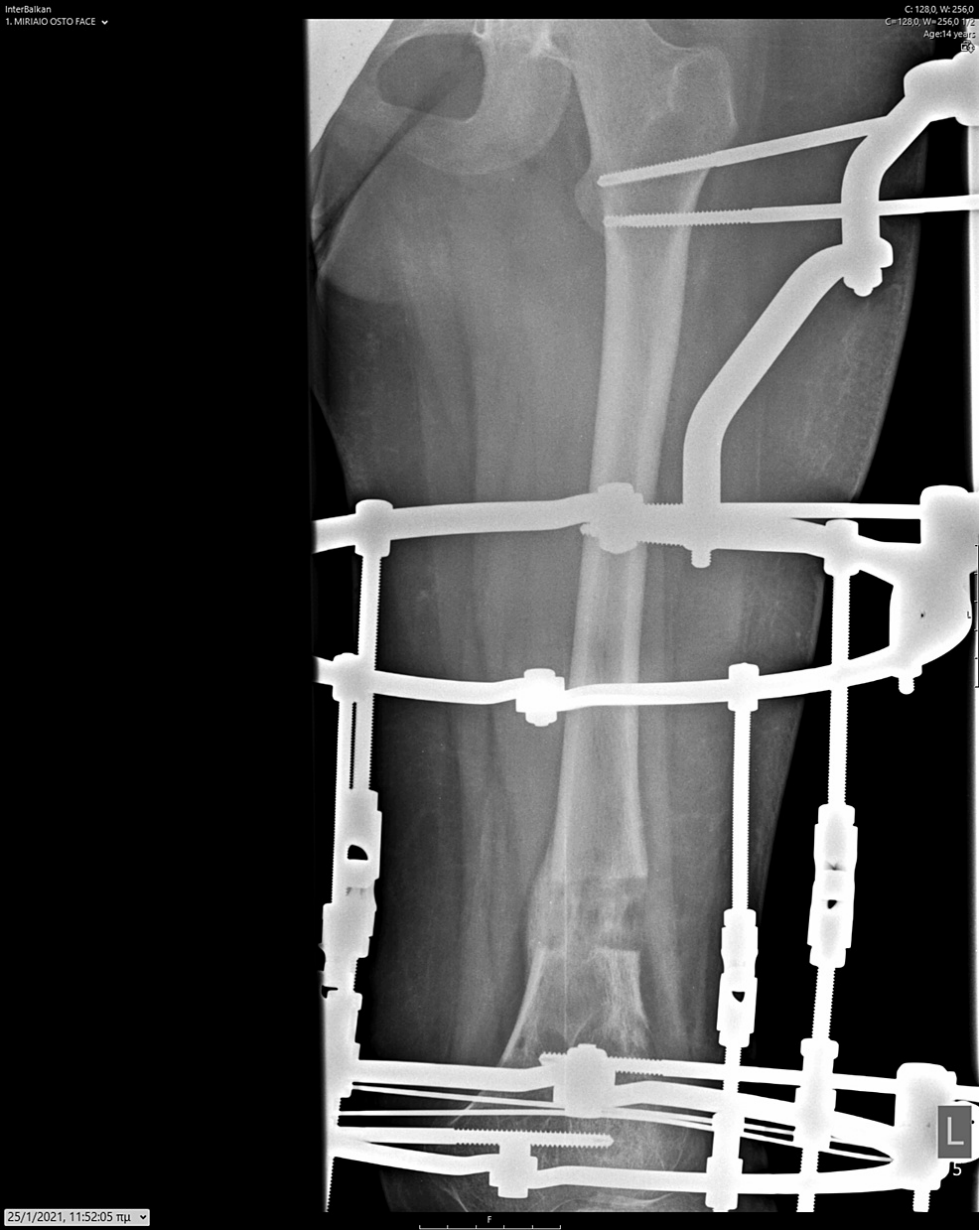
Antero-posterior X-ray at the conclusion of the corrective procedure for femoral valgus deformity, demonstrating adequate calcification of the newly formed bone.

**Figure 14:**
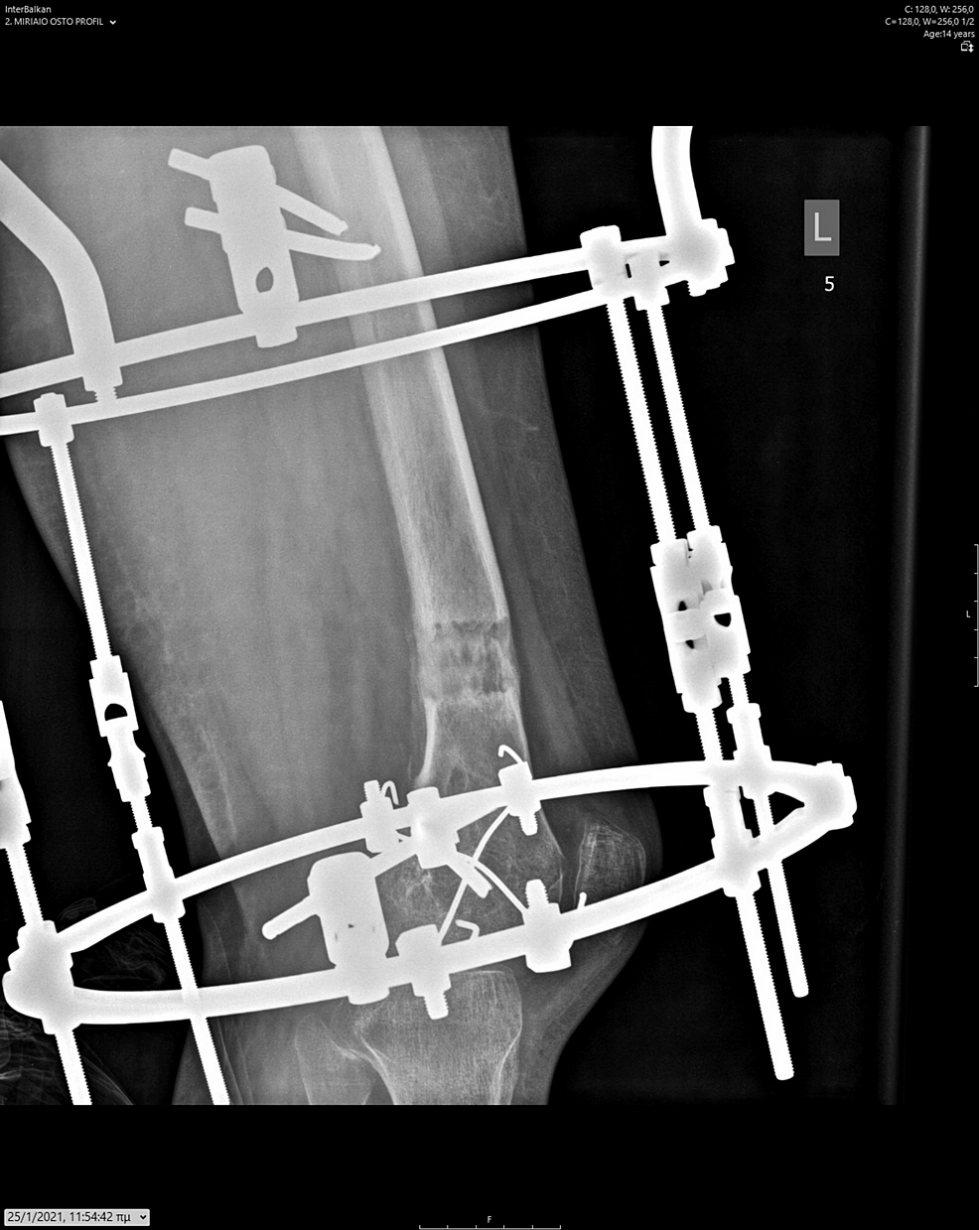
Lateral X-ray at the conclusion of the procedure, displaying appropriate new bone formation.

In accordance with her school attendance, she was surgically treated. We applied a pre-constructed Ilizarov device using a distal ring connected with wires and screws parallel to her knee joint. The central stabilizing ring was connected with two screws, and the central hip ring was connected at the subtrochanteric lesion with two screws. Four lengthening rods were applied. The osteotomy of the distal femoral metaphysis was performed parallel to the distal ring. The lengthening procedure started on the fifth day, using the asymmetry lengthening of the medial to lateral rods. The lateral rods elongated four times a day, while the medial rods elongated twice a day. Her parents were performing the lengthening procedure, and the girl was evaluated every week with appropriate clinical and radiological examination. Pin tracts were regularly cleaned by the parents daily and inspected and further cleaned by us in every clinical attendance. There were no signs of local infection during the whole procedure. After completing the correction of the valgus deformity, as measured with the alignment of the femur and tibia at the knee joint with 10 degrees of valgus, the lengthening procedure was continued with an equal rate of the medial and lateral rays. The patient was walking using crutches with weight bearing as tolerated (Figures 10-14).

**Figure 15:**
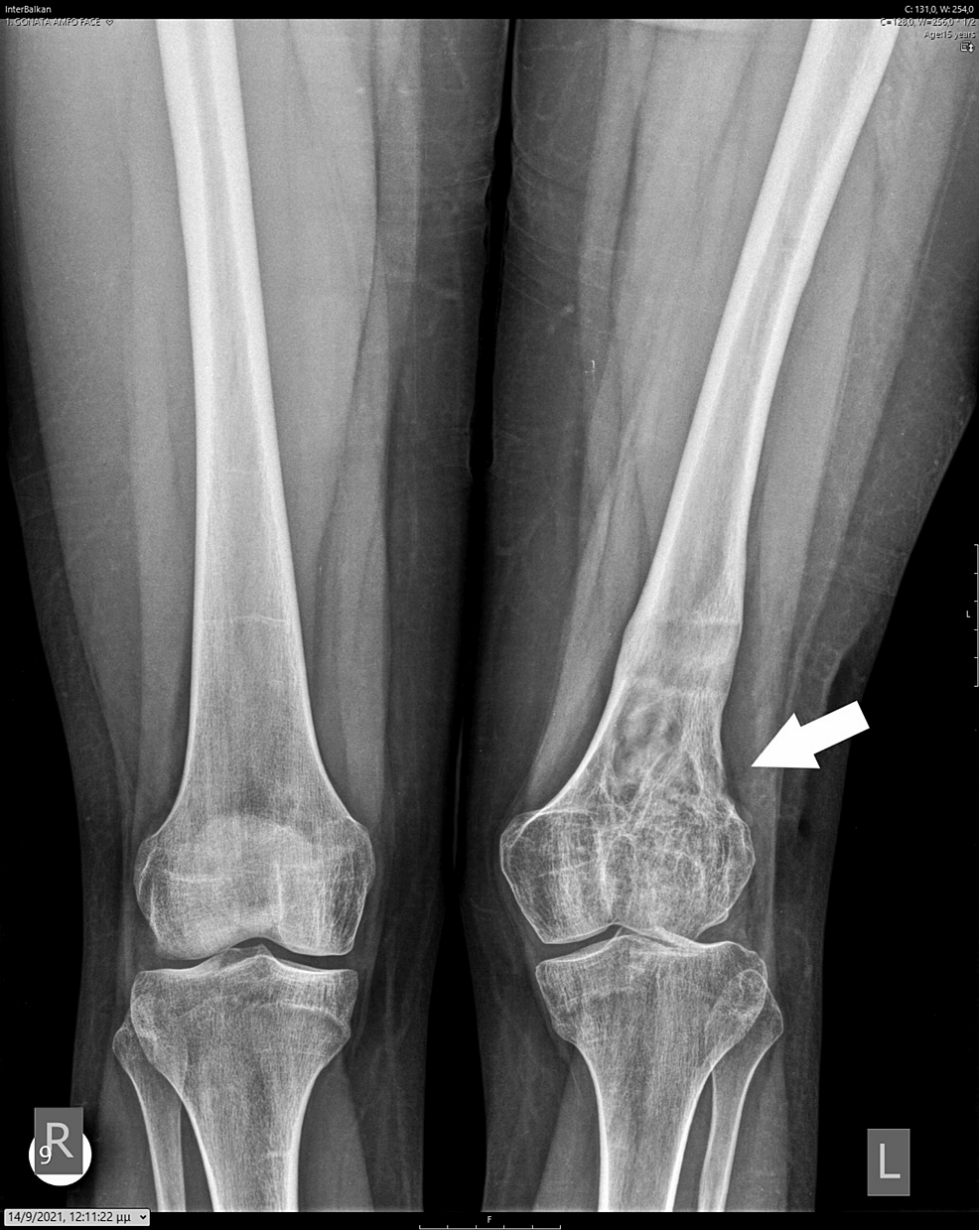
AP X-ray in the standing position, taken one year after the removal of the Ilizarov device, displays adequate bone formation with a residual LLD of 1 cm. AP: Antero-posterior; LLD: Leg length discrepancy.

**Figure 16:**
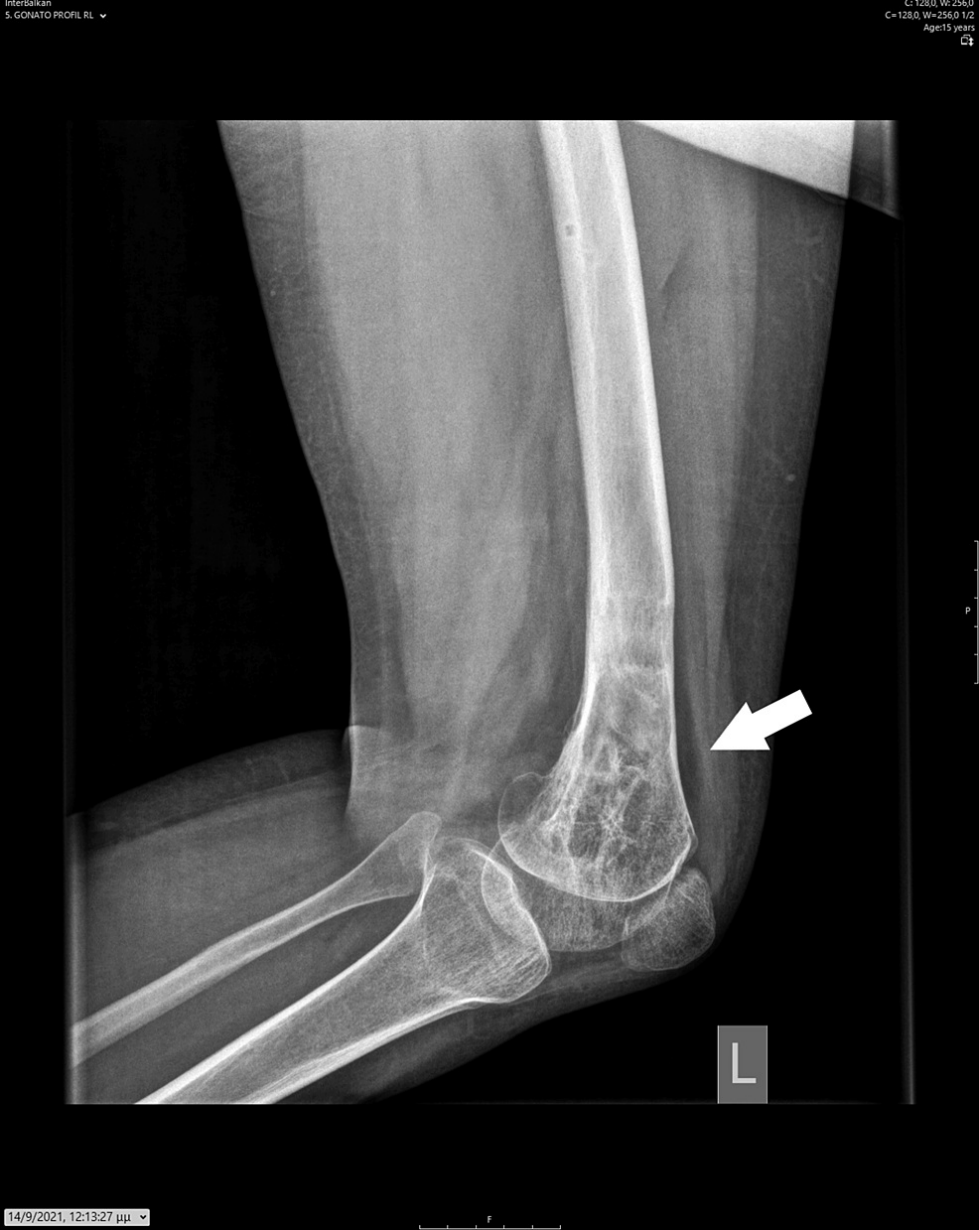
Lateral X-ray of the distal femur, taken one year after the removal of the Ilizarov device.

**Figure 17:**
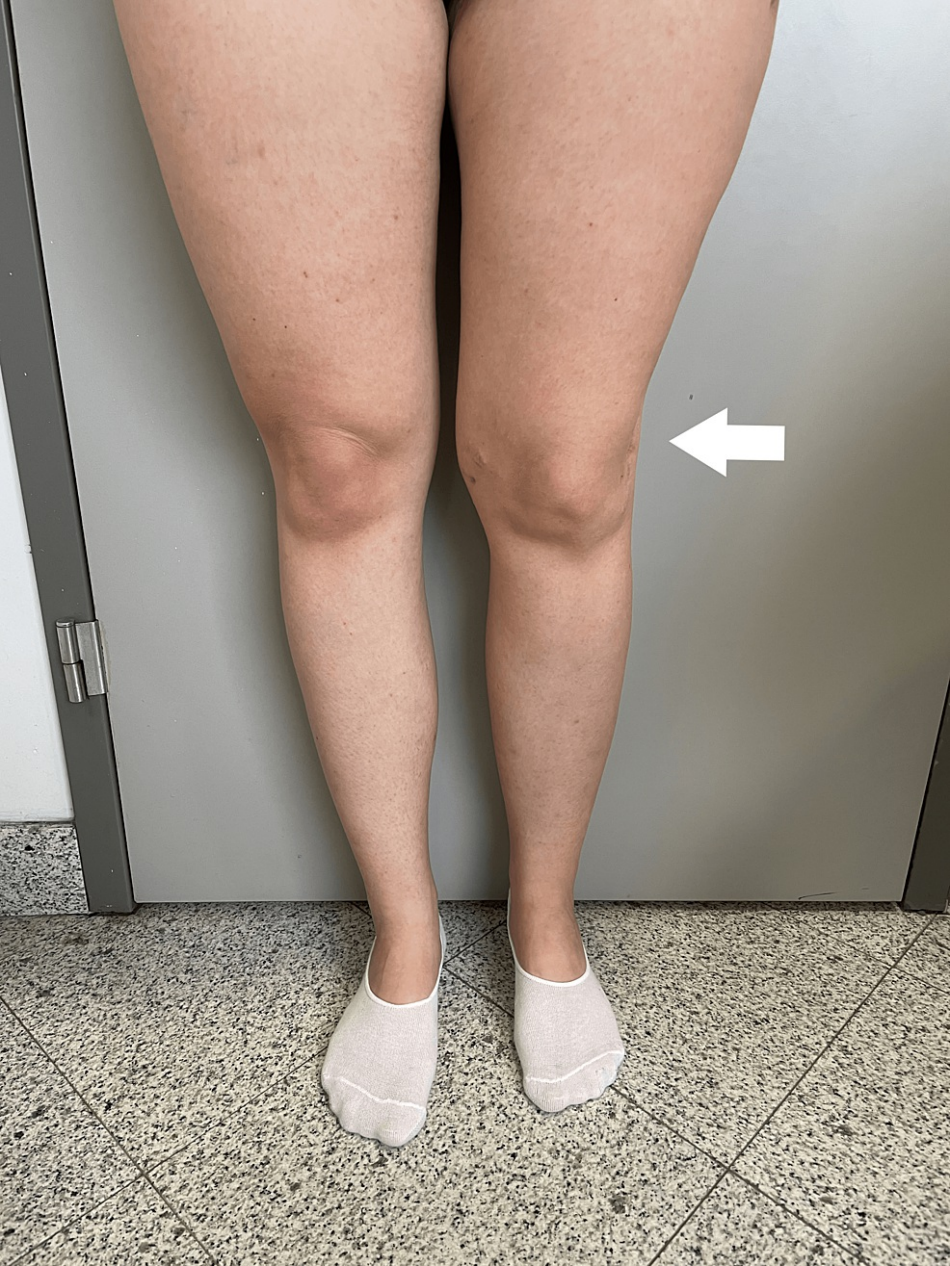
Clinical photograph of the girl standing, illustrating the restoration of limb alignment post-procedure.

New bone formed adequately during the lengthening procedure, showing sufficient calcification of the newly formed tissue. After achieving a 5 cm lengthening over 56 days, the device was locked, and the patient was encouraged to bear full weight and to flex the knee as tolerated. Two months later, upon observing adequate bone formation after partial loosening of the lengthening rods, we removed the Ilizarov device. We prescribed an articulating knee brace for protection and recommended knee exercises and crutch use to protect the newly formed bone. The patient was thrilled with the significant restoration of her leg. After six months of protective measures, we allowed her to walk without crutches. At the most recent follow-up, two years after completing the lengthening procedure, she presents with an LLD of 1 cm, normal axis alignment, and full flexion and extension of the knee. She confidently wears short skirts and expresses great satisfaction with the restored shape of her lower limbs (Figures 15-17).

## Discussion

ABCs are locally aggressive benign bone tumors. Capanna R et al. classified their radiographic appearances into five types [9]. In our case, the cyst, identified as type III, involved the lateral metaphyseal region, impacting only the lateral cortex but penetrating and extending into the epiphysis. There are very few reported cases of ABC penetrating the growth plate and extending into the epiphysis. Cysts are also described as aggressive when they actively and rapidly expand, destroying the cortex. Restrepo R et al. presented a case involving a 14-year-old girl with an expansile metadiaphyseal lesion with epiphyseal involvement. They noted that such cases can sometimes result in growth disturbance and deformity [1, 9, 10]. Lin PP et al. reported that the cyst's juxtaphyseal location is considered an important risk factor for cyst recurrence [1]. In their series, out of 19 patients with juxtaphyseal cysts, eight developed recurrence. When using curettes to clean the cyst, the growth plate is an obstacle to adequately removing the cyst. There is the surgical fear of damaging the physis. However, biological factors may influence the cyst recurrence related to the increased vascularity of the growth plate and the possible production of growth factors. Interestingly, the authors have not found erosions of the growth plate in the follow-up radiological examination. Lampasi M et al. report nine children with ABC of the distal fibula, ages ranging from 7-12, with open physis [11]. Among them, seven cases were juxtaepiphyseal; none was invading the growth plate. They report that the distal fibular growth plate remained active after surgery, and none of the children presented with deformities. However, they mention that juxtaepiphyseal ABC abutting the growth plate may lead to its damage, either by the expansion of the cyst or iatrogenic during curettage [1,11]. The younger age of the initial cyst identification is another crucial risk factor for recurrence. Patients younger than 12 years are reported with higher rates of recurrence [10]. ABCs are reported with a variety of recurrence rates, approximately 12-30%, apart from the type of the initial treatment [1,7]. Döring K et al. reported a recurrence rate of 28 of 90 patients (31%) with ABC, treated surgically with curettage with or without adjuvants or filling [12]. Various treatment methods have been reported. Intralesional curettage with adequate bone grafting remains the standard method. Less invasive methods, such as the intralesional injections of sclerotherapy agents, were popular in previous years. These agents are now primarily used for ABCs that are challenging to access surgically. Alcohol, polidanol, and doxycycline are among the agents still in use [13-17]. However, sclerotherapy with Ethibloc (Ethnor Laboratories, Ethicon, Germany) or H2O2 is associated with serious complications [6,18]. Denosumab, the monoclonal anti-RANK-L antibody, is utilized to manage aggressive ABC cysts in older patients as its use in children is associated with severe complications from hypercalcemia and nephrocalcinosis. Rapid recurrence has been reported after discontinuation of denosumab [19, 20]. Intralesional curettage can be performed through minimally invasive surgery or even with arthroscopic assistance [21, 22].

In our patient with the juxtaphyseal expansion and the intraepiphyseal involvement, using agents that could damage the growth plate was our main consideration, apart from the thorough cleaning of the lesion. We used metaphyseal and epiphyseal openings of the affected lesion, avoiding the damage of the growth plate as much as possible. Our patient experienced satisfactory bone healing post-treatment. The cyst reduced in size, and remarkable cortical regeneration was observed in the bones of the distal femur. Neer CS et al. have proposed a classification system for assessing the healing of the cyst post-treatment, comprising four types [21-24]. Our patient exhibited healing with a radiolucent defect area of less than 50% of the diameter and adequate bone thickness, allowing her to partake in all activities without risk of fracture (Type II, healed cyst with a defect). However, the axial deviation and the LLD became her primary concerns, not recurrence. We have not found reports addressing the management of deformity following the healing of the cyst. Arora S et al. described a case of ABC in the proximal tibia, crossing the open physis, which healed without any defects. They report, based on a literature review, 18 cases of ABC invading the growth plate, with none affecting the distal femoral physis. They suggest that early-stage surgical intervention for juxtaepiphyseal ABC may prevent late-stage disturbances of the growth plate [25]. In our patient, the cyst demonstrated signs of healing, with axis deviation and LLD becoming the primary concerns. We employed the Ilizarov device to concurrently correct both the axis deviation and LLD. The Ilizarov method, through precise placement of hinges and asymmetrical lengthening of the rods, allows for simultaneous correction of axis deviation and lengthening of the affected bone. This method is the preferred choice in cases involving children who have sustained permanent damage to the physis, most commonly due to infection or severe injury of the growth plate [26-28]. By employing an asymmetrical rate, we were able to correct the patient's valgus deformity and LLD.

## Conclusions

We are presenting the long-term outcomes of an ABC that involved the distal femoral metaphysis in a seven-year-old girl, which penetrated the growth plate and extended into the epiphysis. Although the cyst healed, damage to the physis resulted in LLD and valgus axis deviation of the knee upon the completion of the growth period. By employing the Ilizarov apparatus and utilizing asymmetrical lengthening of the rods, we could rectify both the LLD and the axis deviation. The follow-up for an ABC should encompass not only the monitoring of the cyst's healing process but also the restoration of normal limb alignment, particularly when treating ABC in children with open physis. Distraction osteogenesis is an effective method for the management of these deformities.
